# Revaccination

**Published:** 1871-07

**Authors:** 


### REVACCINATION.

The medical profession of England are exercising themselves considerably over the subject of revaccination. Much is being written, both in the medical and secular press, upon this subject, until the public have formed themselves into societies for and against vaccination in any form. Deaths are occurring in London at the rate of about two hundred and fifty per week, and it seems, from the hospital reports, that many have taken place among those who had been vaccinated. For this reason, the physicians urge revaccination, after about thirty years, or during the time of the extensive prevalence of small-pox.

We do not believe that vaccination is a complete protection against smallpox, neither is smallpox impossible in those who have once had the disease, for instances are constantly recurring in persons who have had the regular, confluent form of the disease; yet, we know that vaccination lessens the virulence of the disease, and in the majority of cases acts as a complete protection against its attack. Experience has shown that the protective power of vaccination is limited, and that it should be renewed once in about forty years, or at such times that smallpox may be raging in the community. Those physicians who have had an extended experience with smallpox, particularly in the hospitals of London, consider revaccination the only means of ascertaining the protection retained from the primary operation, and regard the protective influence in direct proportion to the shortness of the time since its performance. As the operation is, in the great majority of cases,—particularly when the virus is taken directly from the cow,—unattended by any danger or inconvenience, we recommend all persons who have passed thirty years since their vaccination, to be revaccinated. If the virus does not take, it is safe to suppose that the influence from the former vaccination has not been lost, and that therefore the protection is as complete as can be afforded.

Occasionally we meet with a case of serious inflammation as a result of vaccination—so do we encounter equal, and even fatal cases, from the accidental scratch of a pin; but, this single case in a thousand, should not be cited as an argument against revaccination. The benefits to be derived from it, through the protection afforded, in the great majority of cases, from a loathsome and dangerous disease, will more than overbalance all the arguments that its opponents may bring against it.

